# *In Situ* Raman and Fourier Transform Infrared Spectroscopy Studies of MXene−Electrolyte Interfaces

**DOI:** 10.1021/acsnano.5c03810

**Published:** 2025-06-09

**Authors:** Tetiana Parker, Yuan Zhang, Kateryna Shevchuk, Teng Zhang, Vikash Khokhar, Young-Hwan Kim, Givi Kadagishvili, David Bugallo, Manushree Tanwar, Ben Davis, Jongyoun Kim, Zahra Fakhraai, Yong-Jie Hu, De-en Jiang, Dmitri V. Talapin, Yury Gogotsi

**Affiliations:** 1 A.J. Drexel Nanomaterials Institute, 6527 Drexel University, Philadelphia, Pennsylvania 19104, United States; 2 Department of Materials Science and Engineering, 6527 Drexel University, Philadelphia, Pennsylvania 19104, United States; 3 Interdisciplinary Materials Science, 5718 Vanderbilt University, Nashville, Tennessee 37235, United States; 4 Department of Chemistry, 2462 The University of Chicago, Chicago, Illinois 60637, United States; 5 Department of Chemistry, 6572 University of Pennsylvania, Philadelphia, Pennsylvania 19104, United States; 6 CiQUS, Universidade de Santiago de Compostela, Santiago 15782, Spain; 7 Department of Chemical and Biomolecular Engineering and Department of Chemistry, 5718 Vanderbilt University, Nashville, Tennessee 37235, United States

**Keywords:** MXene, electrochemical energy storage, *in situ* spectroscopy, Raman spectroscopy, Fourier transform infrared spectroscopy

## Abstract

A comprehensive understanding of electrochemical interfaces is essential for the optimal performance of electrocatalysts, supercapacitors, and batteries. However, understanding the electrochemical behavior of MXenes during electrochemical processes by any single technique does not provide a whole picture. We achieved real-time monitoring in the complete near-mid-infrared chemical range by utilizing Raman spectroscopy (near-infrared (NIR) excitation) and Fourier transform infrared (FTIR) spectroscopy in the mid-infrared (MIR) range. The change of intramolecular O−H vibrations of MXene-confined water was monitored in real time using FTIR, while surface terminations were monitored by using Raman spectroscopy. The dynamic interplay between charge storage and the change in MXene surface chemistry was studied by employing representative electrolytes (0.5 M H_2_SO_4_, 1 M LiCl, and 6 M KOH) and comparing hydrophilic Ti_3_C_2_T*_*x*_* with mixed-terminations (T = O/OH/F) with hydrophobic chlorine-terminated Ti_3_C_2_Cl_2_ MXene electrodes. *Ab initio* molecular dynamics (MD) simulations and density functional theory (DFT) calculations were used to shed light on ion insertion with a dynamic change of ion solvation and reveal the structure of the MXene-confined water.

## Graphical abstract

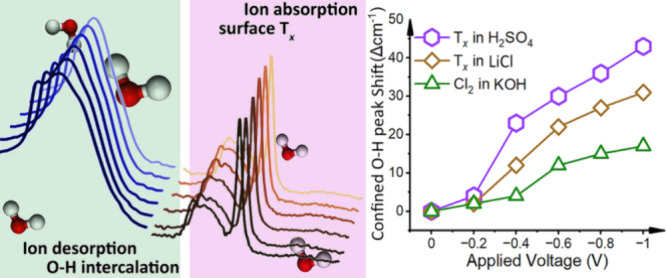

## Introduction

Electrocatalysis and electrochemical energy storage have the potential to address the growing global energy demand and environmental concerns.^1^ A deep understanding of the underlying electrochemical processes has been deemed imperative to optimize device performance. Traditionally, these processes have been analyzed *ex situ*,^2,3^ which limited insights into real-time behavior due to a lack of understanding of the dynamic mechanisms. However, *in situ* techniques have recently been implemented to shed light on the electrochemical processes. For example, vibrational spectroscopies such as Fourier transform infrared (FTIR) and Raman spectroscopy have been employed to analyze the electrochemical reduction of graphene oxide films in organic solvents.^4^ While these techniques are powerful individually, their combination provides complementary information, as FTIR probes change in dipole moment, whereas Raman tracks changes in polarizability. This distinction means that certain molecular vibrations will be selectively active in either Raman or FTIR spectroscopy. The synergistic use of both techniques offers a deeper understanding of novel two-dimensional (2D) material systems including MXenes.

MXenes, a novel class of 2D transition metal carbides, nitrides, and carbonitrides (M_*n*+1_X_*n*_T*_*x*_*, where M is a transition metal, X is carbon or nitrogen, and T*_*x*_* is surface termination (e.g., = −O, −F, −OH, and halogens)),^5^ have emerged as promising materials for electrochemical applications due to their unique properties, including high conductivity,^6^ tunable surface chemistry,^7^ and large surface area.^8^ MXenes have been explored as active materials in supercapacitors^9^ and batteries,^10^ wearable applications,^11^ infrared (IR) shielding,^12^ electromagnetic interference (EMI) shielding,^13^ conductive binding additives,^14^ and as current collectors.^15^

*In situ* studies of 2D materials, such as MXenes, have used specialized electrochemical cells. Confined cells, often employed with Raman spectroscopy, are constructed using materials such as glass or Kapton for cell encapsulation.^16^ Integrated cells, frequently utilized with FTIR spectroscopy, are directly connected to the attenuated total reflectance (ATR) crystal.^7,17,18^ Previous *in situ* studies with other 2D materials, such as graphene^4^ and transition metal dichalcogenides,^19^ have focused on tracking various changes. Chemical transformations, such as electrochemical hydrogen generation and storage,^20,21^ and nitrogen (N_2_) sorption in metal−organic frameworks (MOFs) have been investigated.^22^ Catalytic reactions of the cobalt oxide catalyst^23^ have also been monitored. *In situ* studies of MXenes have revealed crucial insights into their electrochemical behavior. Water evolution has been observed during various electrochemical reactions, such as intercalation and deintercalation of ions.^7,17^ Many studies have also been focused on *in situ* characterization of structural^18^ and optical changes^24^ during electrochemical ion intercalation. However, the complex interplay among the MXene surface chemistry, structural evolution during ion intercalation, and solvent changes caused by ion desolvation during electrochemical reactions is yet to be better understood. To bridge this knowledge gap, this study introduces an *in situ* characterization approach combining Raman and FTIR spectroscopy to monitor the electrochemical behavior of MXenes in real time. By probing both NIR and MIR spectral regions, we have simultaneously tracked changes in intercalants and surface terminations during electrochemical cycling.

This study investigates the dynamic relationship between the charge storage and the adsorption of active species in aqueous electrolytes [sulfuric acid (0.5 M H_2_SO_4_), lithium chloride (1 M LiCl), and potassium hydroxide (6 M KOH)] with varying pH values. This research focuses on two types of MXenes: mixed-terminated Ti_3_C_2_T*_*x*_* and chlorine-terminated Ti_3_C_2_Cl_2_. The primary goal is to understand how the hydrophilicity and hydrophobicity of the MXene surface affect charge storage and solvent dynamics in various aqueous electrolytes. FTIR spectroscopy is employed to analyze intramolecular vibrations, such as hydrogen bonding and O−H stretching vibrations in water. FTIR spectroscopy provides valuable insights into the strength and nature of hydrogen bonds, which are crucial for many electrochemical processes.^25^ Raman spectroscopy is also utilized, as it is sensitive to changes in polarizability and effectively probes the vibrational modes of surface terminations, making it an ideal technique for studying MXene systems.^3,26^

To further study the underlying mechanisms, *ab initio* molecular dynamics (AIMD) simulations have been employed to investigate the behavior of confined water interacting with both ions and the MXene surface. Density functional perturbation theory (DFPT) calculations were performed on representative AIMD snapshots to obtain the change of vibrational signatures with the electrolytes and surface terminations of Ti_3_C_2_T*_*x*_* compared to those of Ti_3_C_2_Cl_2_. AIMD, DFT, and DFPT results confirmed the experimental findings, providing a robust theoretical framework for interpreting the observed electrochemical phenomena. Additionally, atomic force microscopy (AFM) was used to study structural changes. Vibrational peak positions between selected Ti_3_C_2_T*_*x*_* and Ti_3_C_2_Cl_2_ electrodes and vibrational fingerprints of Ti_3_C_2_T*_*x*_* compared to Ti_3_C_2_Cl_2_ were recorded using both Raman and FTIR, providing a comprehensive analysis of electrode materials (Figure 1).

The study combines experimental and theoretical techniques to provide critical insights into the complex interfacial behavior of MXenes.^27^ The insights will enable the rational design and optimization of high-performance, stable MXene-based electrochemical devices, paving the way for a sustainable energy future.

**Figure 1:**
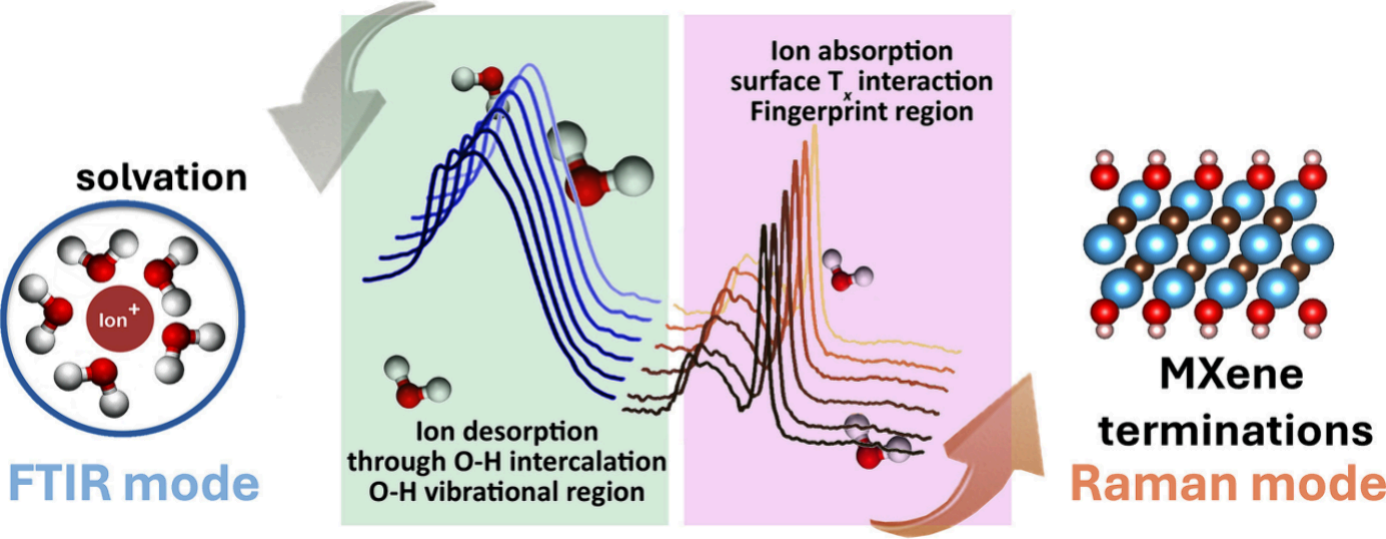
Concept of MXene *in situ* characterization. Combining FTIR and Raman spectroscopy enables comprehensive analysis of ion and MXene-confined water changes and an in-depth understanding of surface termination changes.

## Results and Discussion

### *In Situ* Electrochemical Measurements

To comprehensively investigate the electrochemical behavior of MXenes, three conventional electrode−electrolyte systems were chosen, which were characterized by fundamentally different chemistries. The systems selected include Ti_3_C_2_T*_*x*_* in 0.5 M H_2_SO_4_, Ti_3_C_2_T*_*x*_* in 1 M LiCl, and Ti_3_C_2_Cl_2_ in 6 M KOH, exhibiting acidic and basic electrolytes and mixed and chlorine terminations on the MXene surface, respectively. While exploring all six possible combinations of two MXene types and three electrolytes that would provide a more exhaustive data set, this study focused on demonstrating *in situ* tracking of the most representative electrode−electrolyte pairs to illustrate key electrochemical behaviors. Future work will benefit from broader parametric studies; however, the present work prioritizes showcasing significant differences using representative members of the electrode−electrolyte pairs. Before *in situ* spectroscopic analysis, cyclic voltammetry (CV) was performed (Figure 2a−c) on each system to evaluate the electrochemical stability and potential-dependent behavior of the MXene electrodes.

The CV curves revealed significant variations across the different electrolyte systems, highlighting the strong influence of the electrolyte on the electrochemical response of the MXenes. Ti_3_C_2_T*_*x*_* in H_2_SO_4_ displayed a pseudocapacitive curve with prominent redox peaks (Figure 2a). The redox peaks, observed at −0.6 V vs Ag, are attributed to reversible proton surface termination reactions occurring on the MXene surface.^28^ In contrast, Ti_3_C_2_T*_*x*_* in LiCl exhibited characteristic capacitive behavior, consistent with previous reports in the literature (Figure 2b).^29^ The most intriguing observation was the behavior of Ti_3_C_2_Cl_2_ in 6 M KOH (Figure 2c). This system exhibited a pair of sharp peaks at −0.9 and −1.1 V, a phenomenon not previously reported for Ti_3_C_2_Cl_2_ or other MXenes in the base electrolytes. The unique electrochemical response suggests a distinct charge storage mechanism in the system, likely influenced by the more hydrophobic surface of Ti_3_C_2_Cl_2_ compared to that of Ti_3_C_2_T*_*x*_*. To understand the charge storage mechanism within this system, both surface chemistry and changes in intercalants should be analyzed.

For *in situ* FTIR measurements (Figure 2d,e), the MXene working electrode was positioned directly onto an ATR crystal, ensuring efficient infrared light transmission and maximizing the interaction between the incident infrared beam and the MXene film. For optimal signal quality, Kapton encapsulation of the cell and a sample holder were employed to apply gentle pressure to the cell and maintain consistent contact between the MXene film and the ATR crystal.^2,7,17,18^ For *in situ* Raman spectroscopic analysis (Figure 2f,g), the electrochemical cell was confined within transparent polyethylene terephthalate (PET) films. A hole was punctured at the measurement site to provide an unobstructed Raman signal collection while maintaining a controlled electrochemical environment.^30^

Both FTIR and Raman *in situ* cells presented here are characterized by their simplicity and affordability. Moreover, versatile cell designs can be easily adapted to accommodate various experimental conditions. The setup flexibility allows investigation of a wide range of electrochemical systems, making the *in situ* technique readily accessible and efficient for a broad spectrum of data measurements.

**Figure 2:**
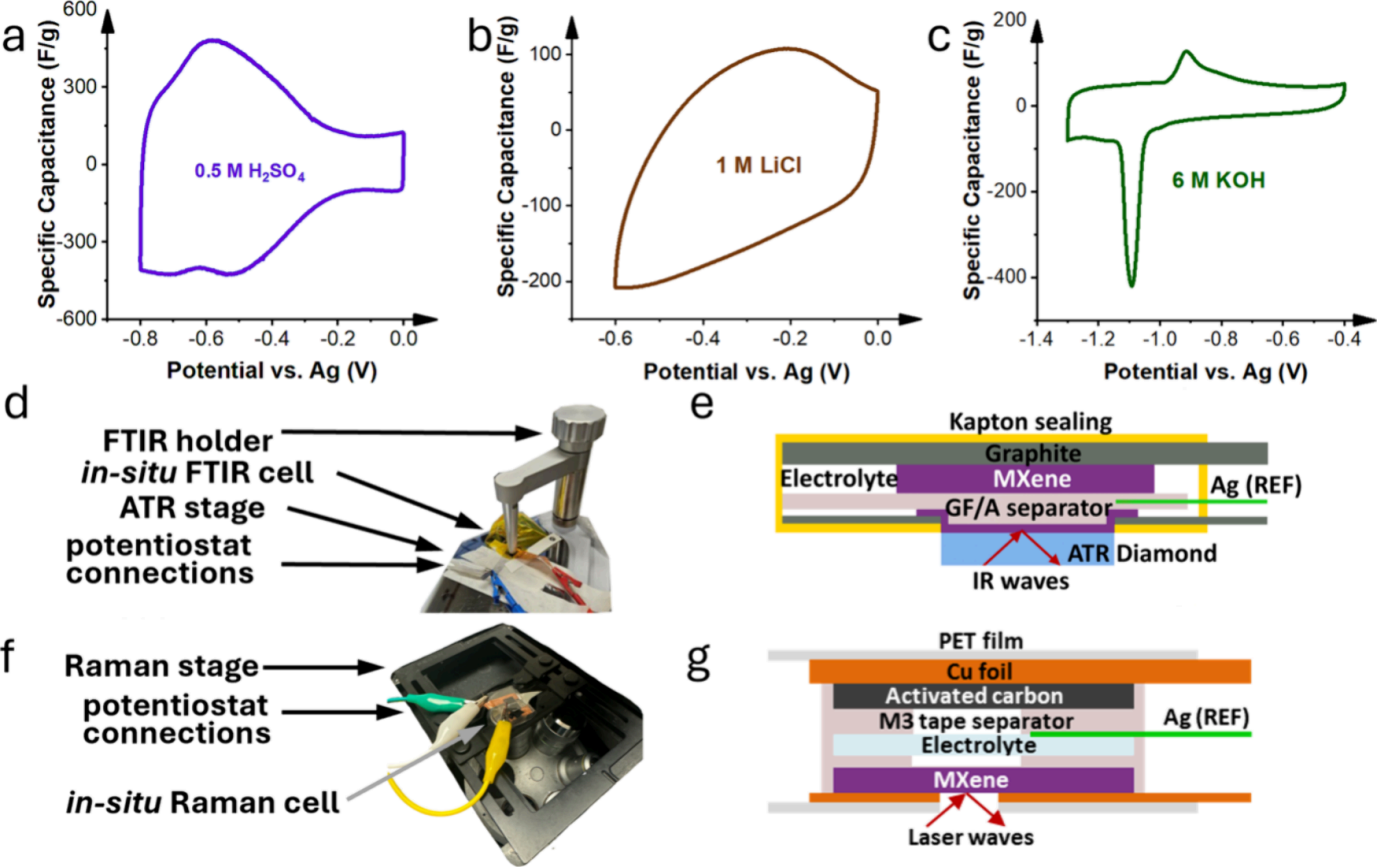
Concept of MXene electrochemical monitoring. (a) Cyclic voltammetry of Ti_3_C_2_T_*x*_ in 0.5 M H_2_SO_4_, (b) cyclic voltammetry of Ti_3_C_2_T_*x*_ in 1 M LiCl, (c) cyclic voltammetry of Ti_3_C_2_Cl_2_ in 6 M KOH, (d) FTIR cell setup, (e) FTIR cell structure schematic (f) Raman cell setup, and (g) Raman cell structure schematic.

### Chemical Characterization

A multifaceted approach was employed to delve into the chemical and structural properties of synthesized Ti_3_C_2_T_*x*_ and Ti_3_C_2_Cl_2_ MXenes. This involved the utilization of FTIR spectroscopy, Raman spectroscopy, and AFM. Additionally, DFT calculations were used to predict and validate the fingerprint region of the MXenes, providing a comprehensive understanding of their molecular structure and vibrational properties.

FTIR spectroscopy provides valuable insights into the vibrational modes of Ti_3_C_2_T*_*x*_* (Figure 3a) and Ti_3_C_2_Cl_2_ (Figure 3b) MXenes, offering clues about their chemical composition and structural properties. The Ti_3_C_2_T*_*x*_* MXene, with its diverse surface terminations (=O, −F, and −OH), exhibited a prominent A_2u_ bending mode in the 600−400 cm^−1^ region. This spectral signature is characteristic of the =O, −F, and −OH functional groups (Tables S1,^2^ S2,^2^ S3). In contrast, the Cl-terminated Ti_3_C_2_Cl_2_ MXene displayed a more pronounced E_u_ stretching mode in the 800−600 cm^−1^ region, confirming the dominance of −Cl terminations on its surface (Table S4). Raman spectroscopy further complemented the FTIR analysis by providing insights into the vibrational modes of the Ti_3_C_2_T*_*x*_* (Figure 3c) and Ti_3_C_2_Cl_2_ (Figure 3d) MXene samples. The Raman spectrum of Ti_3_C_2_T*_*x*_* (Figure 3c) aligns with previously reported results, exhibiting three distinct regions: low-frequency vibrations of the whole flake, followed by vibrational modes from mixed surface terminations, and higher frequency vibrations dominated by carbon atoms.^26^ The combination of in-plane and out-of-plane modes from heterogeneously distributed =O, −F, and −OH groups results in peak broadening, which was previously confirmed by MD simulations.^31^ Therefore, it is unsurprising that Ti_3_C_2_ MXene, with homogeneously distributed chlorine terminations, shows three narrow bands (Table S4 and Figure 3d). They correspond to out-of-plane vibrations: the first peak at 173 cm^−1^ belongs to A_1g_ (Ti, C, Cl), while the 390 and 645 cm^−1^ peaks are related to the A_1g_ (Cl) and A_1g_ (C) modes, respectively. Additionally, the intensity of the peaks depends on the excitation wavelength,^32^ which was maintained at 785 nm in all experiments to ensure consistency.

**Figure 3:**
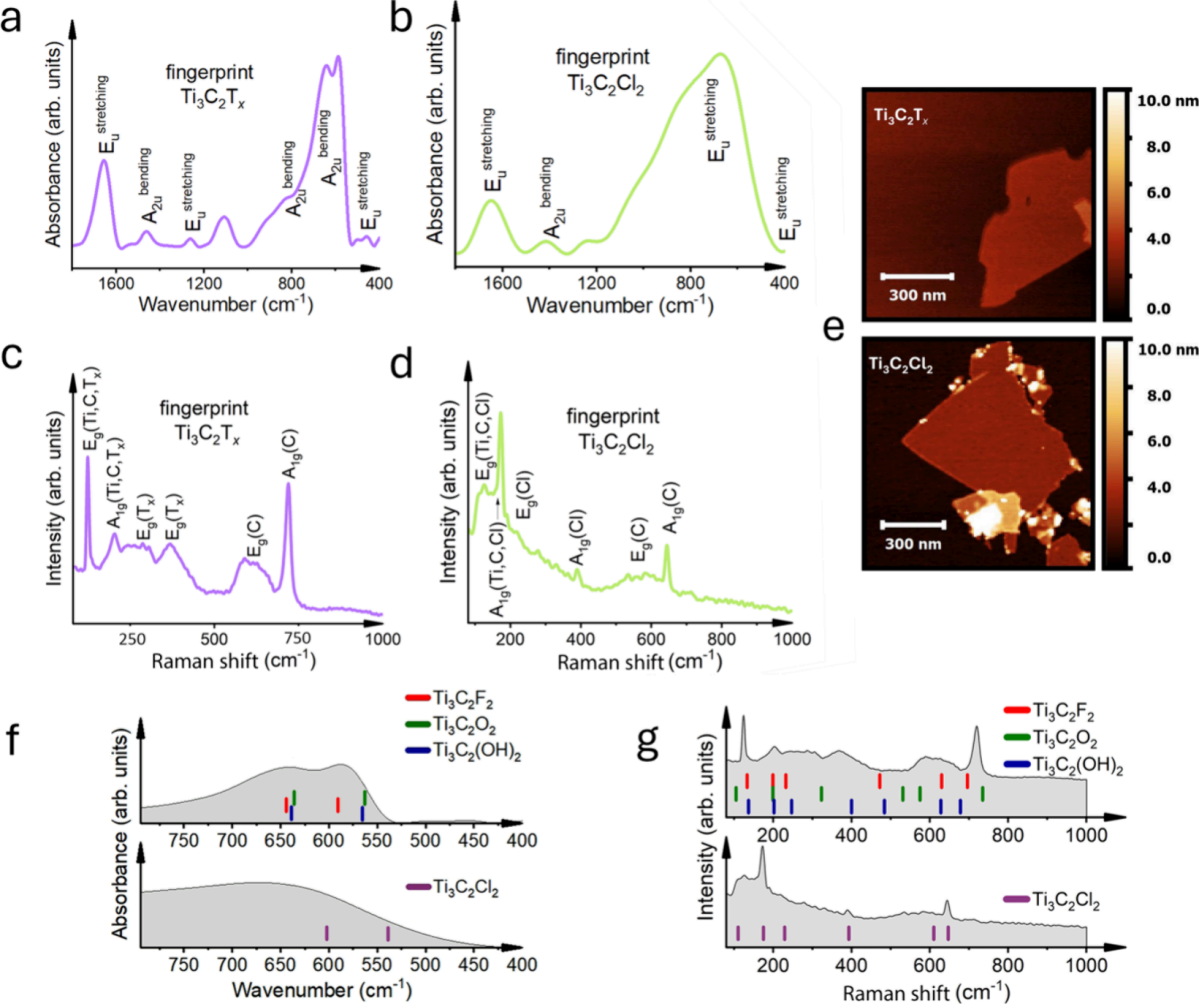
Chemical characterization of Ti_3_C_2_T_*x*_ and Ti_3_C_2_Cl_2_. (a) FTIR fingerprint of pristine Ti_3_C_2_T_*x*_, (b) FTIR fingerprint of pristine Ti_3_C_2_Cl_2_, (c) Raman fingerprint of pristine Ti_3_C_2_T_*x*_, (d) Raman fingerprint of pristine Ti_3_C_2_Cl_2_, (e) AFM images of Ti_3_C_2_T_*x*_ (top) and Ti_3_C_2_Cl_2_ (bottom) on the silicon substrate, (f) IR active vibrational peaks (bars represent DFT predicted IR vibrational peak positions, and gray areas represent experimental data of pristine Ti_3_C_2_T_*x*_ and Ti_3_C_2_Cl_2_), and (g) Raman active vibrational modes (bars represent DFT predicted Raman peak positions, and the gray areas represent experimental spectra of pristine Ti_3_C_2_T_*x*_ and Ti_3_C_2_Cl_2_).

AFM analysis revealed some morphological differences between the MXene samples (Figure 3e). The single-layer MXene flake heights on the Si substrate, calculated from height distribution images as shown in Figure S5, are similar: 2.2 ± 0.3 nm for Ti_3_C_2_T*_*x*_* and 2.1 ± 0.2 nm for Ti_3_C_2_Cl_2_. Ti_3_C_2_Cl_2_ flakes had a more pronounced tendency to form larger, aggregated structures, as seen, for example, as brighter regions in the lower portion of the image. This stacking of multiple MXene flakes affects the overall morphology and uniformity. This disparity can be attributed to either stronger interlayer interactions in the Cl-terminated MXene or inefficient delamination. These morphological differences can potentially impact some applications, influencing factors such as electrical conductivity,^6^ surface area,^8^ and ion diffusion rates.^1^ Ti_3_C_2_T*_*x*_* synthesis has been extensively optimized,^33^ and further optimization for Ti_3_C_2_Cl_2_ can potentially improve the single flake morphology, which is beyond this manuscript. In addition, some small white dots are also observed in Ti_3_C_2_Cl_2_, which may be TiO_2_ nanoparticles, salt,^34^ or impurities resulting from the delamination process. However, we note that TiO_2_ was not observed in the FTIR spectra. These flake heights are comparable to the previously reported Ti_3_C_2_T*_*x*_* flake height of 1.7 ± 0.1 nm measured from the substrate.^34^

DFT calculations were performed to better understand the observed experimental results and simulate the vibrational spectra of both Ti_3_C_2_T*_*x*_* and Ti_3_C_2_Cl_2_, predicting the IR (Figure 3f) and Raman active (Figure 3g) vibrations. For Ti_3_C_2_T*_*x*_*, various termination models (=O, −F, and −OH) were considered (Tables S1,^2^ S2,^2^ S3), while for Ti_3_C_2_Cl_2_, a pure Cl-terminated model (Table S4) was selected. The calculated vibrational frequencies and intensities for IR and Raman spectra agreed with the experimental data (Figure 3f,g). The broad experimental spectra result from numerous peaks of mixed terminations, as predicted for Ti_3_C_2_T*_*x*_*. In Figure 3f, DFT predictions indicate the presence of peaks at approximately 600 and 540 cm^−1^ for Ti_3_C_2_Cl_2_, which are not sharply resolved in the experimental data. DFT predictions estimate singular vibration positions from idealized models, unlike experimental IR profiles, which accumulate intensities across a broad range. This results in a broader experimental spectral profile due to multiatom interactions. A broad peak observed in the experimental Ti_3_C_2_Cl_2_ fingerprint region confirms this expected broadening. By combination of FTIR and Raman spectroscopy, a more comprehensive understanding of the vibrational properties of MXenes can be gained, aiding in the optimization of their synthesis and applications in various fields.

### *In Situ* Spectroscopy

To systematically investigate the impact of electrochemical potential on the chemical properties of MXene systems, a constant voltage holding (CstV) protocol was employed (Figure S1). The protocol involved a stepwise increase in voltage with a 2 min hold at each step for FTIR and Raman spectra recording. Applying the controlled potential bias induced specific electrochemical charge storage states in the MXene electrodes, enabling the study of the resulting chemical transformations. The approach allowed for a detailed examination of the relationship among applied potential, surface chemistry, and the overall electrochemical behavior of the MXenes.

FTIR spectroscopy (Figure 4a) revealed significant changes in the intramolecular vibrational region of the O−H^17^ stretching (3700−2800 cm^−1^) during the CstV experiment, particularly for the H_2_SO_4_ electrolyte. The changes are attributed to the dynamic interplay between weak charge-induced O−H stretching, which is water attracted by MXene surface oxygen terminations (in the frequency range of 3700−3500 cm^−1^ in the case of T*_*x*_* and 3600−3200 in the case of Cl_2_ terminations), and stronger cation-induced O−H stretching, that is, the water molecules that surround the cations (in a vibrational frequency range of 3500−3200 cm^−1^ in the case of T*_*x*_* and 3200−2800 in the case of Cl_2_ terminations) within the MXene nanoconfinement. The intensity and positions of the O−H peaks were sensitive to the potential-induced charge-surface interaction (Figure 4g,h). Under negative potential, a stronger interaction between the charge and the surface terminations was induced, which led to the redshift of both confined water peaks. In addition, the proton charge-induced O−H stretching peak intensity decreased with more negative potential, which implies the strengthening of the intermolecular hydrogen bonding network.^35^ This indicates that the electrochemical state of MXene influences the hydrogen bonding network and the degree of water confinement. The above suggests that the electrolyte ions and applied potential can significantly affect the hydration environment and the interfacial properties of the MXene electrodes.

In the LiCl system, the behavior of confined and solvated O−H groups during the CstV experiment was also probed using *in situ* FTIR spectroscopy (Figure 4b). The O−H stretching region exhibited distinct spectral features that evolved with the applied potential. The intensity and position of these peaks were sensitive to the degree of water confinement within the MXene interlayers (Figure 4g,h). The O−H stretching bands in the frequency range of 3700−3500 cm^−1^ associated with Li^+^-confined water molecules dominated at lower potentials (0 V). As the potential increased (nearing −1 V), the intensity of these bands increased, while the intensity of bands associated with MXene-confined water molecules decreased. This suggests that the applied potential influences the distribution of water molecules within the MXene nanoconfinement. The redistribution of water molecules within the MXene confinement indicates ion desolvation, which can impact the ion transport properties and electrochemical performance of the MXenes.

Moreover, the Cl-terminated MXene (Figure 4c) exhibited a lower O−H band absorbance relative to that of the fingerprint region, suggesting the absence of hydroxyl groups on the surface. This observation aligns with the hydrophobic nature of the electrode.^32^ This hydrophobicity can also be observed from the FTIR spectra in the water region (3600−2800 cm^−1^). With the Cl-terminations, the accessibility of water molecules is greatly suppressed.^32^ The difference in surface chemistry can significantly impact the interfacial properties of MXenes, influencing their electrochemical behavior and stability. Ti−O vibrations were observed in the 650−550 cm^−1^ region^2^ for T*_*x*_*-terminated Ti_3_C_2_T*_*x*_* systems immersed in both 0.5 M H_2_SO_4_ and 1 M LiCl electrolytes. Conversely, the C−Cl vibrations were detected in the 850−650 cm^−1^ region^36,37^ for Ti_3_C_2_Cl_2_ in 6 M KOH.

*In situ* Raman spectroscopy with 0.5 M H_2_SO_4_ revealed the most prominent changes in peak positions compared to those of other electrolyte systems (Figure 4d, Figure S6). The shifts of the in-plane E_g_ (Ti, C, T*_*x*_*) mode (peak at 123 cm^−1^) (Figure S6a,d) to higher wavenumbers and the out-of-plane A_1g_ (C) mode (peak at 720 cm^−1^) (Figure S6h,k) to lower wavenumbers with decreasing potential were observed, confirming the protonation of the surfaces. In this case, peak shifts of 2 cm^−1^ (123.5 to 125.5 cm^−1^) and 6 cm^−1^ (722 cm^−1^ to 716 cm^−1^) were observed, and these shifts are reversible (Figure 4i). This phenomenon has been explored theoretically, showing that an abundance of OH surface terminations leads to the same peak changes we observed.^31^ Additionally, previous *in situ* work by Johnson et al. described a similar 720 cm^−1^ peak position change in aqueous acidic electrolytes, whereas neutral electrolytes did not yield similar changes (in that study, the 123 cm^−1^ peak was not analyzed).^38^ While this change has been attributed to proton absorption on the MXene surfaces, FTIR spectroscopy indicates that water, a weak Raman scatterer, interacts with MXene during electrochemical cycling, potentially leading to O−H bonds on the surfaces that produce Raman peak shifts.

**Figure 4:**
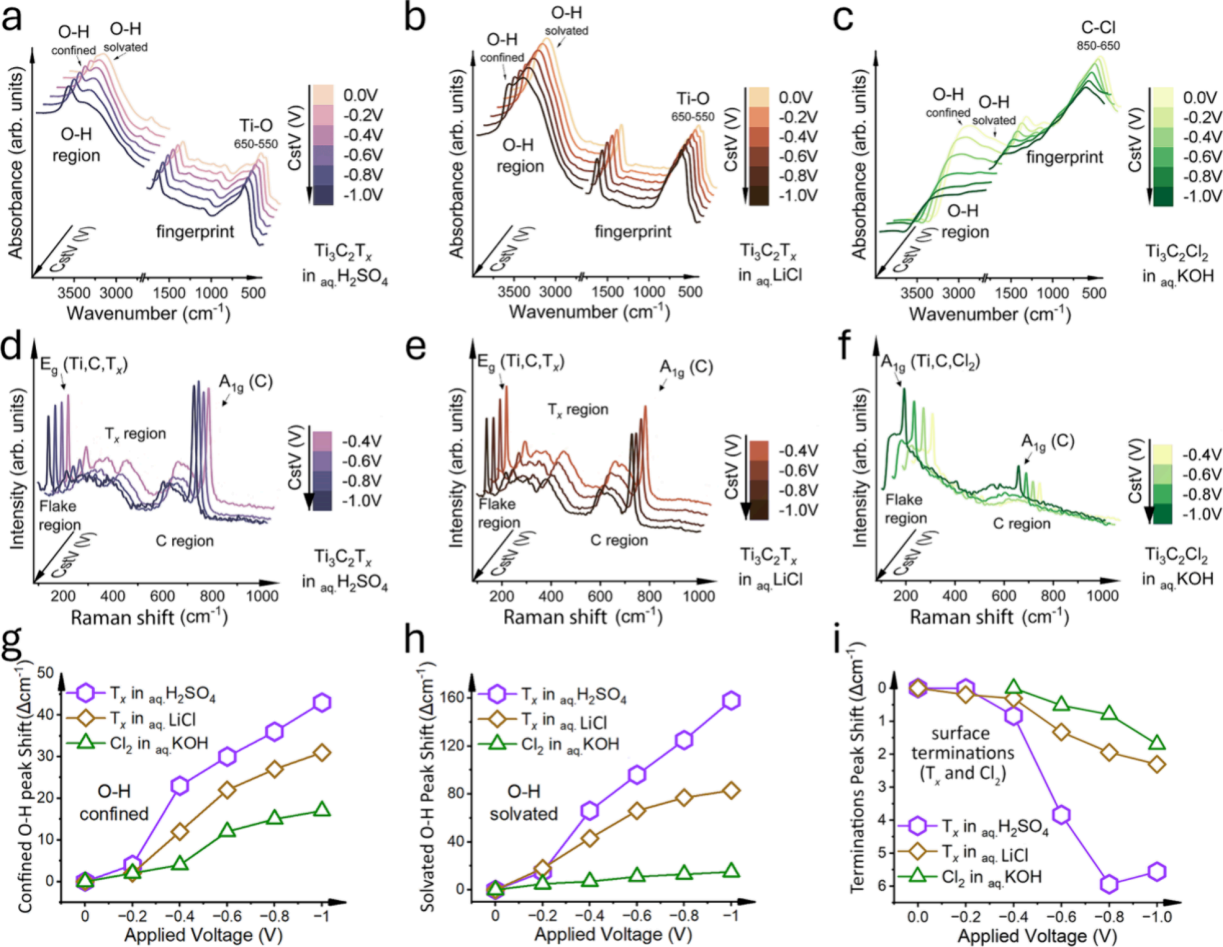
*In situ* near-mid-infrared characterization. (a) FTIR spectra of Ti_3_C_2_T_*x*_ in 0.5 M H_2_SO_4_, (b) FTIR spectra of Ti_3_C_2_T_*x*_ in 1 M LiCl, (c) FTIR spectra of Ti_3_C_2_Cl_2_ in 6 M KOH, (d) Raman spectra of Ti_3_C_2_T_*x*_ in 0.5 M H_2_SO_4_, (e) Raman of Ti_3_C_2_T_*x*_ in 1 M LiCl, (f) Raman spectra of Ti_3_C_2_Cl_2_ in 6 M KOH, (g) confined vibrational peak shift, (h) O−H solvated vibrational peak shift, and (i) termination vibrational peak shift. Given the smaller magnitude of Raman shifts, panels (d−f) illustrate the electrochemical behavior from −0.4 to −1.0 V, where the most significant changes in the material’s structure and composition are caused by electrochemical redox and ion intercalation processes (see panel (i)).

Compared to the H_2_SO_4_ electrolyte, the MXene peak shift in 1 M LiCl is smaller (Figure 4e, Figure S6b,i). The in-plane vibrational mode of the entire flake shifted from 123.6 to 123.2 cm^−1^ (Figure S6f), and the out-of-plane carbon vibration shifted from 720.5 to 718.5 cm^−1^ (Figure S6m). While such small shifts could technically be attributed to a measurement error, the reversibility of these changes suggests their structural or compositional origin. Li^+^-confined water molecules may interact with MXene surfaces, but due to the larger ion size, their contribution to surface terminations is less significant than in the sulfuric acid electrolyte. Additionally, a small shift (1.1 cm^−1^) of the 720 cm^−1^ peak to the lower wavenumber has been previously reported in tensile stress experiments with 0.4% applied strain.^39^ Therefore, the strain associated with insertion of ion into the structure may contribute to the observed peak shifts. This would also explain the shift of the 123 cm^−1^ peak to a lower wavenumber in the LiCl system. It contrasts with sulfuric acid, where the blue shift is indicative of increased O−H content, as shown by MD.^31^ Together with FTIR spectroscopy, these findings suggest a large impact of ion−water molecule confinement in the system.

The electrochemical behavior of Ti_3_C_2_Cl_2_ has not been previously studied *in situ*. We observed changes in the A_1g_ (C) vibrational mode with increasing potential in 6 M KOH (Figure 4f and Figure S6j). The smallest shift of 1.5 cm^−1^, from 645.8 to 644.3 cm^−1^, was noted (Figure 4i). Similar to the H_2_SO_4_ and LiCl systems, this shift may indicate a small contribution of ions to surface terminations. However, since the structure is uniformly terminated with chlorine and is highly stable, this shift is more likely to represent strain within the structure caused by the intercalation of large K^+^ ions. As the contribution of water molecules is dampened in the FTIR spectra, it can be concluded that the structure remains stable in terms of surface terminations during electrochemical cycling.

Combining FTIR and Raman spectroscopy enables a comprehensive understanding of the reaction mechanisms and interfacial processes occurring at the MXene electrode−electrolyte interface. FTIR spectroscopy highlights the intricate interplay of water molecules with the MXene surface, distinguishing between different water environments and providing insights into the hydration and ion desolvation processes. With its precision in tracking vibrational mode shifts, Raman spectroscopy offers direct evidence of structural changes within the MXene lattice, attributed to factors such as ion intercalation/deintercalation, protonation of the surface, and strain induced by ion insertion. This combined approach allows for a deeper understanding of the complex interplay among the electrolyte, the MXene surface, and the electrochemical reactions at the interface. FTIR spectroscopy characterizes the water environment, and Raman spectroscopy provides insights into the structural and chemical transformations within the MXene lattice itself.

### Understanding O−H Behavior

*In situ* FTIR spectroscopy was coupled with AIMD simulations and DFPT calculations to investigate further the complex interplay among the MXene surface, electrolyte ions, and solvent molecules. AIMD simulations provide a detailed atomistic view of the interfacial processes, including ion adsorption, solvation, and hydrogen bonding. By comparing the experimental FTIR spectra with the calculated vibrational spectra from DFPT, insights into the structural and dynamical properties of the MXene−electrolyte interface can be gained. This integrated approach allows for identifying specific molecular interactions, quantifying the degree of ion adsorption, and predicting the impact of different electrolyte compositions on the electrochemical performance of MXenes.

The FTIR spectra revealed significant changes in the O−H stretching region (3600−3400 cm^−1^) during the electrochemical cycling. These changes are attributed to the dynamic behavior of water molecules at the MXene-electrolyte interface, which is influenced by the applied potential and nature of the electrolyte ions. The intensity and position of the O−H bands can be correlated with the degree of water confinement between the MXene surfaces.

With increasing electrolyte pH, the intramolecular O−H stretching of MXene-confined water (3750−3500 cm^−1^) shifts to a lower frequency region (Figure 5a−c), which indicates the weakening of the O−H bonding and stronger interaction between water molecules and MXene surface terminations.

**Figure 5:**
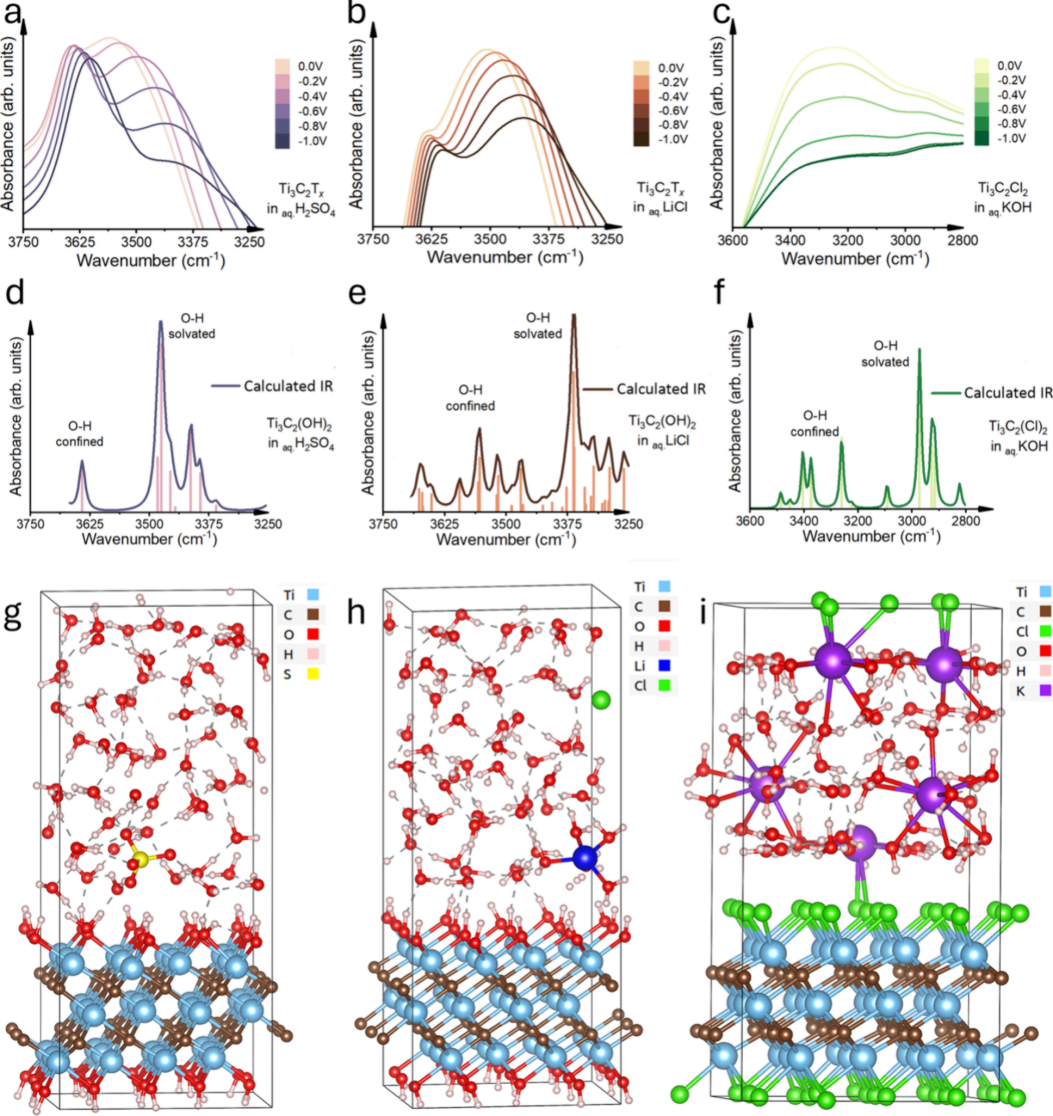
Signatures of the O−H stretching of MXene-confined water. (a) Experimental O−H region of Ti_3_C_2_T_*x*_ in 0.5 M H_2_SO_4_, (b) experimental O−H region of Ti_3_C_2_T_*x*_ in 1 M LiCl, (c) experimental O−H region of Ti_3_C_2_Cl_2_ in 6 M KOH, (d) DFPT-calculated IR spectrum of the O−H region of Ti_3_C_2_T_*x*_ in 1 M H_2_SO_4_, (e) DFPT-calculated IR spectrum of the O−H region of Ti_3_C_2_T_*x*_ in 1 M LiCl, (f) DFPT-calculated IR spectrum of the O−H region of Ti_3_C_2_Cl_2_ in 6 M KOH, (g) a snapshot of AIMD-simulated Ti_3_C_2_T_*x*_ in 1 M H_2_SO_4_ at 5 ps, (h) a snapshot of AIMD-simulated of Ti_3_C_2_T_*x*_ in 1 M LiCl at 5 ps, and (i) a snapshot of AIMD-simulated Ti_3_C_2_Cl_2_ in 6 M KOH at 5 ps.

Under negative potential, all of the peaks in the three systems shifted to a lower frequency range. For Ti_3_C_2_T*_*x*_* in the H_2_SO_4_ electrolyte, a pronounced peak shift to lower frequency is observed, which indicates that a stronger hydrogen bonding is induced under the nuclear quantum effect as more protons are transferred within the confinement.^35^ In the LiCl electrolyte, the low-frequency peak shift is observed due to ion dehydration under applied potential that enhances the ion−water Coulombic force and weakens the O−H stretching vibration in water. For Ti_3_C_2_Cl_2_ in the KOH electrolyte, with a weak hydrogen bonding network in the base electrolyte, the drastic intensity decrease in the peak at a lower frequency (3300−3200 cm^−1^) shows a pronounced ion dehydration effect.

The AIMD snapshots in Figure 5g−i show the interactions between electrolyte ions, MXene layer, and water molecules, with solvation environments and hydrogen bonding. Figure S7 provides a detailed schematic illustrating the differences between solvated and confined −OH groups. The variations in absorbance spectra between Figure 5d and e are attributed to the different electrolytes used, highlighting the electrolytes’ influence on the water hydrogen-bond network and the interfacial behavior. Although such a difference can be already seen to some extent in the snapshots (Figure 5g,h), more in-depth computational modeling and analysis could shed further light in the future. For the present, this study aims to showcase the effectiveness of *in situ* FTIR and Raman spectroscopy as a primary analytical tool, with MD simulations providing supplementary insights. For the H_2_SO_4_ system (Figure 5a,d,g), the experimental and simulated spectra exhibited a strong peak at around 3625 cm^−1^, corresponding to MXene-confined water molecules, and a broader peak at approximately 3500−3375 cm^−1^, attributed to water molecules strongly hydrogen-bonded to the OH-terminated MXene surface. This suggests that the strong interaction with the protonated MXene surface leads to more strongly confined water layers at the MXene surface terminations. For the LiCl system (Figure 5b,e,h), the simulated spectra showed a more intense peak at around 3375 cm^−1^, corresponding to more pronounced Li^+^ charge-induced polarization of water molecules at the ion solvation shells, and a weaker peak at higher frequencies, indicating reduced MXene-confined water interaction. This observation is consistent with the weaker interaction between water and the MXene surface. In the case of the KOH system (Figure 5c,f,i), the simulated spectra revealed a strong and broad peak at a lower frequency range of around 3000 cm^−1^, with the insignificant MXene-confined water peak, caused by the hydrophobicity of the Cl-termination.

## Conclusions

This research, which combines *in situ* Raman and FTIR spectroscopy with DFT calculations and AIMD simulations, opens several important perspectives on the complex electrochemical behavior of MXenes, moving beyond a simple characterization of advanced energy materials. The real-time, near-mid-infrared monitoring enabled by this approach offers a unique window into the dynamic interplay between charge storage and MXene surface chemistry. Specifically, the simultaneous observation of changes in intramolecular O−H vibrations of MXene-confined water (using FTIR spectroscopy) and MXene surface terminations (using Raman spectroscopy) provides unprecedented insight into the behavior of water molecules at the MXene−electrolyte interface and the structural changes within the MXene lattice during electrochemical cycling. The ability to differentiate between hydrophilic and hydrophobic MXene terminations and to observe their distinct electrochemical responses in various electrolytes highlights the crucial role of surface chemistry in the MXene performance. Furthermore, the correlation of experimental data with computational modeling provides a robust framework for interpreting complex phenomena, revealing the intricate relationships among ion intercalation/deintercalation, protonation, strain, and water confinement within the MXene interlayers. The combined experimental and theoretical approach not only deepens the fundamental understanding of MXene interfacial behavior but also lays the groundwork for future investigations of tailored MXene materials for specific electrochemical applications. It offers a powerful methodology for exploring the intricacies of MXene electrochemistry and paves the way for the rational design of next-generation energy storage devices. The *in situ* tracking of interfacial dynamics, particularly the real-time observation of water molecules’ behavior and MXene surface changes, offers a direct pathway to monitoring and optimizing electrolytes and selecting MXene surface terminations for enhanced charge storage. This detailed understanding of hydrophilic and hydrophobic MXene responses, coupled with our robust computational framework, enables precise tailoring of MXene, ultimately facilitating the development of next-generation energy storage devices with improved performance and stability.

## Experimental Section

### MXene Synthesis

Ti_3_C_2_T*_*x*_* and Ti_3_C_2_Cl_2_ were synthesized to investigate their electrochemical properties and interfacial behavior. Ti_3_C_2_T*_*x*_* was synthesized via a conventional wet chemical etching method, employing a mixture of hydrofluoric acid (HF, 50_aq_.%, Sigma-Aldrich) and hydrochloric acid (HCl, 37_aq._%, Sigma-Aldrich) as the etchant. The MAX phase precursor, Ti_3_AlC_2_,^6^ was selected as the starting material for T*_*x*_* and Cl-terminated MXenes. For T_*x*_-terminated MXene preparation, the MAX phase was immersed in the etchant solution and subjected to a controlled temperature and time regimen.^6^ The Al atoms were selectively etched, resulting in the exfoliation of the Ti_3_C_2_T*_*x*_* layers. Subsequent washing and centrifugation were employed to remove the residual etchant and impurities, yielding a multilayered Ti_3_C_2_T*_*x*_*. It was delaminated using LiCl (99%, anhydrous, Sigma-Aldrich). In contrast, Ti_3_C_2_Cl_2_ was synthesized through a molten salt etching of the same MAX phase.^40,41^ The material preparation and etching were performed in an Ar-filled glovebox with oxygen and moisture levels below 1 ppm. The Ti_3_AlC_2_ MAX phase was mixed with CdCl_2_ (99^+^%, anhydrous, Strem) salt in a 1:8 molar ratio. The mixture was heated in an alumina crucible at 610 °C for 6 h. Cl-functionalized MXenes were recovered from the reaction mixture by dissolving excess CdCl_2_ and Cd metal in concentrated aqueous HCl (12.1 M, 37%, fuming, Fisher), followed by washing with deionized water until neutral pH. The resulting MXene was dried under a vacuum at 45 °C for more than 12 h before further use.

### AFM Characterization

AFM measurements were taken to compare the Ti_3_C_2_Cl_2_ and Ti_3_C_2_T*_*x*_* MXene monolayers. The samples were prepared by spin coating (500 rpm for 60 s, 2000 rpm for 10 s). 100 μL of a dilute solution of each MXene was placed onto a Si wafer, which was cleaned for 10 min under oxygen plasma before spin coating. Afterward, a Bruker Icon AFM instrument was used to explore the surface in tapping mode. An AFM probe with a tip radius of ∼10 nm and a resonance frequency of ∼300 kHz (Tap300Al-G) was purchased from Budget Sensors Company. AFM images were acquired with a resolution of 512 samples per line. In the postanalysis, Gwyddion software was applied using the median of differences method to flatten the background. Height histograms shown in Figure S5 were generated after the flattening process and were used to calculate the flake thickness as the distance between the distribution of the substrate and that of the first flake. The full width at half-maximum for each distribution was used to estimate the error in thickness for each MXene.

### DFT Modeling

Density functional theory (DFT) calculations within the Vienna *Ab initio* Simulation Package (VASP)^42^ were employed to investigate the electronic and vibrational properties of MXenes. The projector-augmented wave (PAW)^43^ method was utilized, and the Perdew−Burke−Ernzerhof (PBE)^42^ generalized gradient approximation (GGA) function was adopted to describe electron exchange-correlation. A vacuum layer of approximately 10 Å was introduced to eliminate interactions between the 2D MXene slab and its periodic images. For the phonon calculations, the Brillouin zone was sampled by using a 6 × 6 × 1 Γ-centered k-point mesh, and a plane wave energy cutoff of 600 eV was employed. Phonon calculations were conducted by using density functional perturbation theory (DFPT) within a 4 × 4 × 1 supercell. The obtained phonon dispersion bands (Figure S2)^2^ were corrected using the rotational sum rule,^43^ and the infrared and Raman-active vibrational modes were predicted using the Phonopy Spectroscopy package.^44^

### *In Situ* Spectroscopy Setup

To enable real-time monitoring of the electrochemical processes at the MXene−electrolyte interface, a custom-built *in situ* electrochemical cell was designed to accommodate FTIR and Raman spectroscopy. A three-electrode setup with a silver (Ag) reference electrode was used in both cases. For *in situ* FTIR spectroscopy, a thin film of MXene was attached directly to the ATR crystal, which served as the working electrode. An oversized MXene film and Ag wire were applied as the counter and reference electrodes. A Whatman (GF/A) glass fiber membrane was used as a separator. The electrochemical cell was designed to minimize interference of cell components and ensure optimal optical alignment. An FTIR spectrometer (Bruker Invenio R) was employed to collect the vibrational spectra of the MXene electrode at various stages of the electrochemical cycle. Concave rubberband correction and 25-point average smoothing were applied to the FTIR spectra. The 2700−2000 cm^−1^ region was excluded from testing data as it only shows the ATR crystal signal.^2^ *In situ* Raman spectroscopy was conducted using a Renishaw inverted inVia Raman spectrometer equipped with a 785 nm laser. A 63× objective lens (NA = 0.7), acquisition times of 30−60 s, and low laser powers were employed to prevent laser ablation. The *in situ* cell was assembled by placing a free-standing MXene film onto a PET film, followed by copper foils as the current collectors, a silver wire as the reference electrode, and an activated carbon counter electrode. The cell was sealed and insulated using 3M tape, with an electrolyte opening filled with filter paper during assembly. The electrolyte was introduced into the cell by using a syringe, and the assembled cell was secured on the Raman stage with tape. A Biologic SP-300 potentiostat was connected to each cell for electrochemical data recording simultaneously with FTIR and Raman spectra acquisition.

### Electrochemical Testing

A Biologic SP-300 potentiostat was applied in this study. A constant voltage holding (CstV) protocol (Figure S1) induced specific electrochemical states in the MXene electrodes. The voltage was increased stepwise from 0 V to a predetermined value of −1 V vs Ag wire, with a fixed holding time of 2 min at each step of 0.2 V. This protocol allowed for a systematic investigation of the impact of electrochemical potential on the chemistry of MXenes and the aqueous solution confined between MXene nanosheets. During the CstV experiment, a constant potential was applied to the working electrode, while the current response was monitored over time. The electrochemical cell was held for enough time to stabilize the induced current, and the system could reach a steady state, enabling the *in situ* spectroscopic techniques to capture the chemical changes at the electrode−electrolyte interface.

### AIMD Modeling

Density functional theory (DFT) calculations with the Vienna *Ab initio* Simulation Package (VASP) were performed for structure optimization.^45^ PBE exchange-correlation functional and PAW pseudopotential were applied.^46–48^ Plane-wave cutoff energy was 500 eV, and atomic positions were relaxed until a force convergence of 0.01 eV Å^−1^. Grimme’s DFT-D3 method was used for vdW corrections with zero-damping.^49^ The Brillouin zone was sampled with a K-points grid 3 × 3 × 1 for surface calculation.^50^ *Ab initio* molecular dynamics simulations (AIMD)^51^ were performed with a Nosé−Hoover thermostat^52^ in NVT ensemble, with a time-step of 1 fs and constant velocities at 300 K. AIMD equilibrated structures, at 300 K for 5 ps, were used for density functional perturbation theory (DFPT)^53^ calculations to generate IR spectra using the code from Karhánek.^54^

12-unit supercell structures of Ti_3_C_2_(OH)_2_ and Ti_3_C_2_Cl_2_ were used as MXene surfaces. The −OH termination was chosen as Ti_3_C_2_T*_*x*_* representation because our previous AIMD simulations have shown that the −OH and -O terminations on the MXene surface are interconvertible via proton transfer with water,^55^ while −F terminations are rather inert.^56^ So, the Ti_3_C_2_(OH)_2_ termination is a reasonable starting configuration, and future work will examine additional representations of the surface terminations. A random arrangement of water molecules and electrolyte ions was chosen on MXene surfaces with approximate concentrations of 1 M H_2_SO_4_ (Figure 5g), 1 M LiCl (Figure 5h), and 6 M KOH (Figure 5i) solutions, reasonably close to experimental values while saving computational cost. This resulted in three simulated systems (Figure 5, Figure S3): Ti_3_C_2_(OH)_2_ + 1 M H_2_SO_4_, Ti_3_C_2_(OH)_2_ + 1 M LiCl, and Ti_3_C_2_Cl_2_ + 6 M KOH. Extensive equilibration and convergence checks were performed on the chosen initial configurations to validate its accuracy within the study’s scope. As a test, three different initial structures were chosen for Ti_3_C_2_(OH)_2_ in the 1 M LiCl model and the simulated radial distribution functions of Li^+^−O_water_, averaged over 3−5 ps, were similar (Figure S4), indicating that the water dynamics in these systems are fast enough for our AIMD simulations to reach equilibrium and sufficiently sample the configurations.

## Supplementary Material



## Data Availability

The original data that support the findings of this study are available from the authors upon request.

## Notes

DFT vibrational positions; constant voltage holding (CstV) protocol; phonon dispersion bands; *ab initio* molecular dynamics simulations (AIMD) data; radial distribution functions of Li^+^−O_water_; atomic force microscopy (AFM) flake height distribution; Raman spectral peak position and intensity changes; comparison of OH-confined vs OH-solvated systems (PDF)
