# Supplementary material for: In Situ Raman and Fourier Transform Infrared Spectroscopy Studies of MXene−Electrolyte Interfaces
Source: ACS Nano. 2025 Jun 9;19(24):22228–39. doi: 10.1021/acsnano.5c03810 (PMC12203640; doi:10.1021/acsnano.5c03810)
Supplement: Supplementary file 1 [file nn5c03810_si_001.pdf]

# Supporting Information

# *in situ* Raman and Fourier-Transform Infrared Spectroscopy Study of MXene-Electrolyte Interfaces

Tetiana Parker,[#,1,2] Yuan Zhang,[#,1,2] Kateryna Shevchuk, [#,1,2] Teng Zhang,[1,2] Vikash Khokhar,[3] Young-Hwan Kim,[4] Givi Kadagishvili,[5] David Bugallo,[2,6] Manushree Tanwar,[5] Ben Davis,[1,2] Jongyoun Kim,[1,2] Zahra Fakhraai,[*,5] Young-Jie Hu,[*,2] De-en Jiang,[*,3,7] Dmitri V. Talapin,[*,4] Yury Gogotsi,[*,1,2]

[#]These authors contributed equally to the work
*Corresponding authors. Email: fakhraai@sas.upenn.edu, yh593@drexel.edu, de-en.jiang@vanderbilt.edu, dvtalapin@uchicago.edu, gogotsi@drexel.edu

[1]A.J. Drexel Nanomaterials Institute, Drexel University, Philadelphia, PA 19104, USA
[2]Department of Material Science and Engineering, Drexel University, Philadelphia, PA 19104, USA
[3]Interdisciplinary Materials Science, Vanderbilt University, Nashville, TN 37235, USA
[4]Department of Chemistry, The University of Chicago, Chicago, IL 60637, USA
[5]Department of Chemistry, University of Pennsylvania, Philadelphia, PA 19104, USA
[6]CiQUS, Universidade de Santiago de Compostela, Santiago 15782, Spain
[7]Department of Chemical and Biomolecular Engineering and Department of Chemistry, Vanderbilt University, Nashville, TN 37235, USA

**Table S1**. Density functional theory (DFT) vibrational positions predicted for $Ti_3C_2O_2$

| v [cm$^{-1}$] | Ir. Rep. | I [e$^2$amu$^{-1}$] |
|---|---|---|
| -0.000008 | None | 0.000000 |
| 130.987473 | $E_g$ | 0.000000 |
| 189.682975 | $A_{1g}$ | 0.000000 |
| 205.816684 | $E_u$ | 0.039091 |
| 332.158025 | $E_u$ | 15.942252 |
| 338.826370 | $E_g$ | 0.000000 |
| 362.340129 | $A_{2u}$ | 0.101889 |
| 490.907524 | $E_u$ | 4.437631 |
| 545.061738 | $E_g$ | 0.000000 |
| 562.565738 | $A_{1g}$ | 0.000000 |
| 563.255136 | $A_{2u}$ | 0.592402 |
| 636.042683 | $A_{2u}$ | 0.080401 |
| 739.142393 | $A_{1g}$ | 0.000000 |

**Table S2**. DFT vibrational positions predicted for $Ti_3C_2F_2$

| v [cm$^{-1}$] | Ir. Rep. | I [e$^2$amu$^{-1}$] |
|---|---|---|
| 0.000001 | None | 0.000000 |
| 141.854725 | $E_g$ | 0.000000 |
| 198.398782 | $A_{1g}$ | 0.000000 |
| 226.668469 | $E_u$ | 1.013951 |
| 240.231082 | $E_g$ | 0.000000 |
| 278.138529 | $E_u$ | 0.052498 |
| 339.864898 | $A_{2u}$ | 0.067846 |
| 456.941725 | $A_{1g}$ | 0.000000 |
| 464.034181 | $A_{2u}$ | 0.027895 |
| 591.164478 | $A_{2u}$ | 0.177249 |
| 628.664565 | $E_g$ | 0.000000 |
| 643.651564 | $E_u$ | 48.477369 |
| 691.508969 | $A_{1g}$ | 0.000000 |

**Table S3**. DFT vibrational positions predicted for $Ti_3C_2(OH)_2$

| v [cm$^{-1}$] | Ir. Rep. | I [e$^2$amu$^{-1}$] |
|---|---|---|
| -0.000015 | None | 0.000000 |
| 140.909956 | $E_g$ | 0.000000 |
| 203.709424 | $A_{1g}$ | 0.000000 |
| 236.786577 | $E_u$ | 0.446726 |
| 255.975162 | $E_g$ | 0.000000 |
| 278.166062 | $E_u$ | 0.847518 |
| 356.704734 | $A_{2u}$ | 0.007445 |
| 407.449822 | $E_g$ | 0.000000 |
| 409.046901 | $E_u$ | 0.530931 |
| 479.668848 | $A_{1g}$ | 0.000000 |
| 486.612844 | $A_{2u}$ | 0.044941 |
| 565.142616 | $A_{2u}$ | 0.006911 |
| 627.510540 | $E_g$ | 0.000000 |
| 639.054138 | $E_u$ | 5.637117 |
| 673.044673 | $A_{1g}$ | 0.000000 |
| 3680.878825 | None | 6.420879 |

**Table S4**. DFT vibrational positions predicted for $Ti_3C_2Cl_2$

| v [cm$^{-1}$] | Ir. Rep. | I [e$^2$amu$^{-1}$] |
|---|---|---|
| 0.000003 | None | 0.000000 |
| 112.945304 | $E_g$ | 0.000000 |
| 163.153753 | $A_{1g}$ | 0.000000 |
| 183.797913 | $E_u$ | 0.149571 |
| 214.784249 | $E_g$ | 0.000000 |
| 270.899955 | $E_u$ | 0.090799 |
| 273.827512 | $A_{2u}$ | 0.054828 |
| 376.398433 | $A_{1g}$ | 0.000000 |
| 411.849347 | $A_{2u}$ | 0.281332 |
| 538.672285 | $A_{2u}$ | 0.569902 |
| 593.826348 | $E_g$ | 0.000000 |
| 601.849948 | $E_u$ | 9.428776 |
| 643.492495 | $A_{1g}$ | 0.000000 |

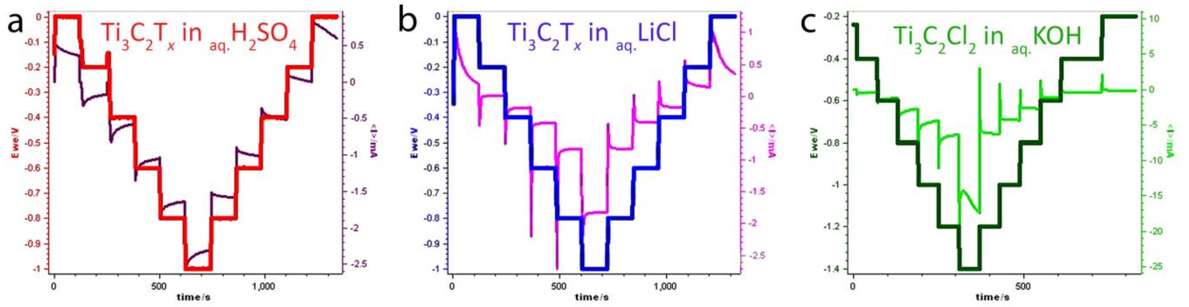


**Figure S1.** Constant Voltage holding (CstV) protocol. a) CstV of $Ti_3C_2T_x$ in 0.5 M $H_2SO_4$, b) CstV of $Ti_3C_2T_x$ in 1 M LiCl, c) CstV of $Ti_3C_2Cl_2$ in 6 M KOH.

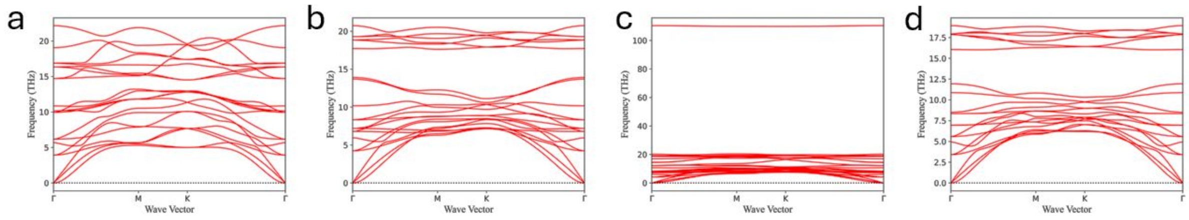


**Figure S2.** Phonon dispersion bands. a) $Ti_3C_2F_2$, b) $Ti_3C_2O_2$, c) $Ti_3C_2(OH)_2$, d) $Ti_3C_2Cl_2$.

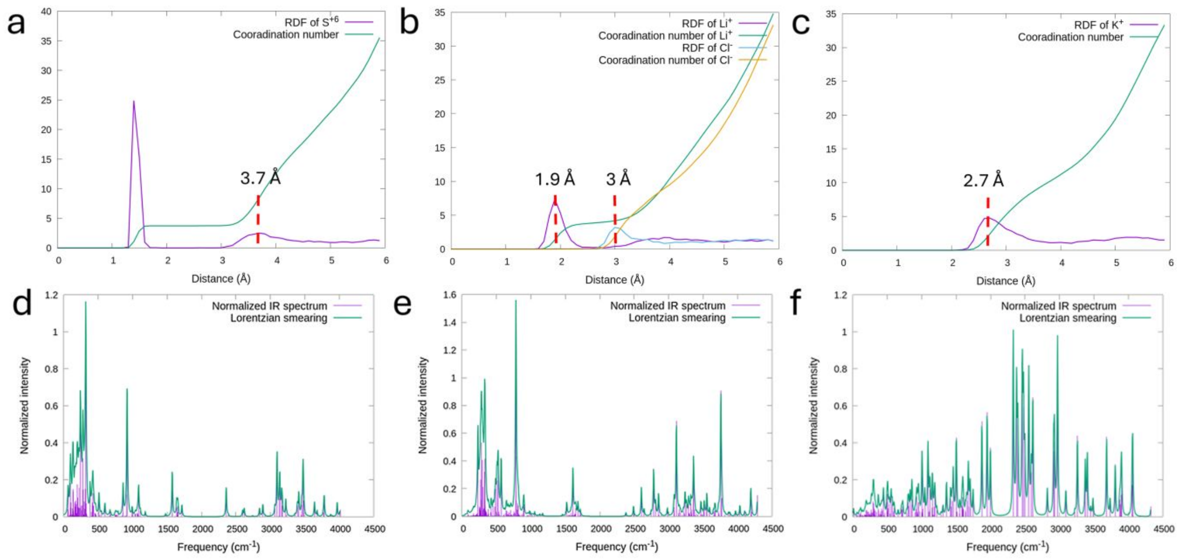


**Figure S3.** *Ab initio* molecular dynamics Simulations (AIMD) data. a) Radial distribution function (RDF) of $S^{+6}$ – O of water for $Ti_3C_2(OH)_2$ in 1 M $H_2SO_4$, b) RDF of $Li^+$ – O and $Cl^-$ – O for $Ti_3C_2(OH)_2$ in 1 M LiCl, c) RDF of $K^+$ – O for $Ti_3C_2Cl_2$ in 6 M KOH, d) DFPT generated IR spectrum of $Ti_3C_2(OH)_2$ in 1 M $H_2SO_4$, e) DFPT generated IR spectrum of $Ti_3C_2(OH)_2$ in 1 M LiCl, f) DFPT generated IR spectrum of $Ti_3C_2Cl_2$ in 6 M KOH.

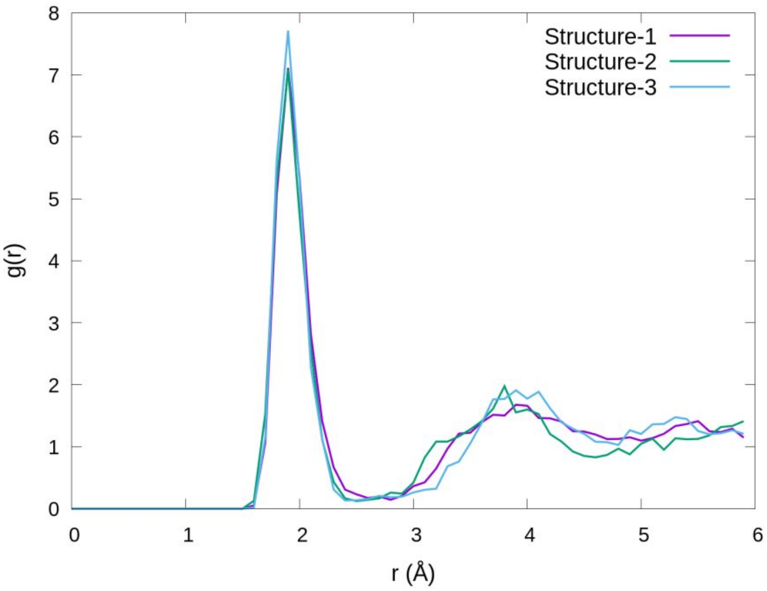


**Figure S4.** Radial distribution functions of $Li^+$ – $O_{water}$ for three different initial structures of $Ti_3C_2(OH)_2$ in 1 M LiCl, averaged over 3-5 ps of ab initio molecular dynamics simulations.

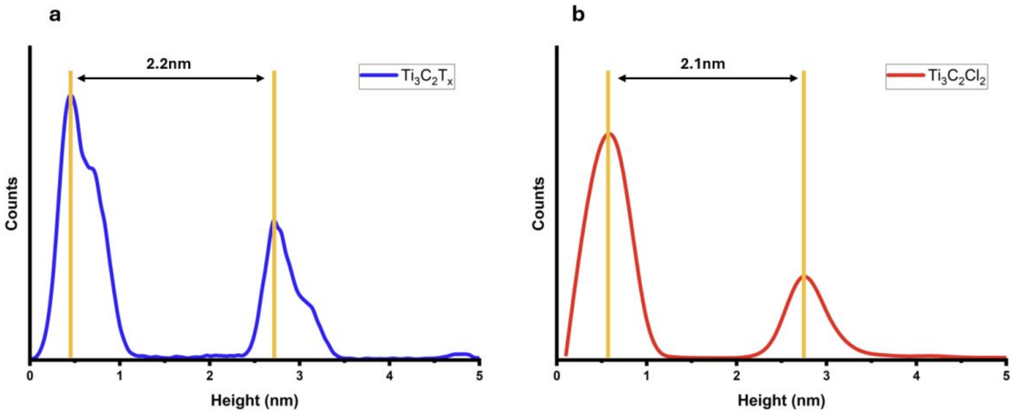


**Figure S5.** Atomic Force Microscopy (AFM) flake height distribution for (a) $Ti_3C_2T_x$ and (b) $Ti_3C_2Cl_2$. In both images, the first distribution originates from the silicon substrate and the second distribution originates from the first MXene layer, with the difference reflecting the average height of MXene from the substrate (shown by double arrows), measured to be 2.2± 0.3 nm and 2.1± 0.2 nm, respectively.

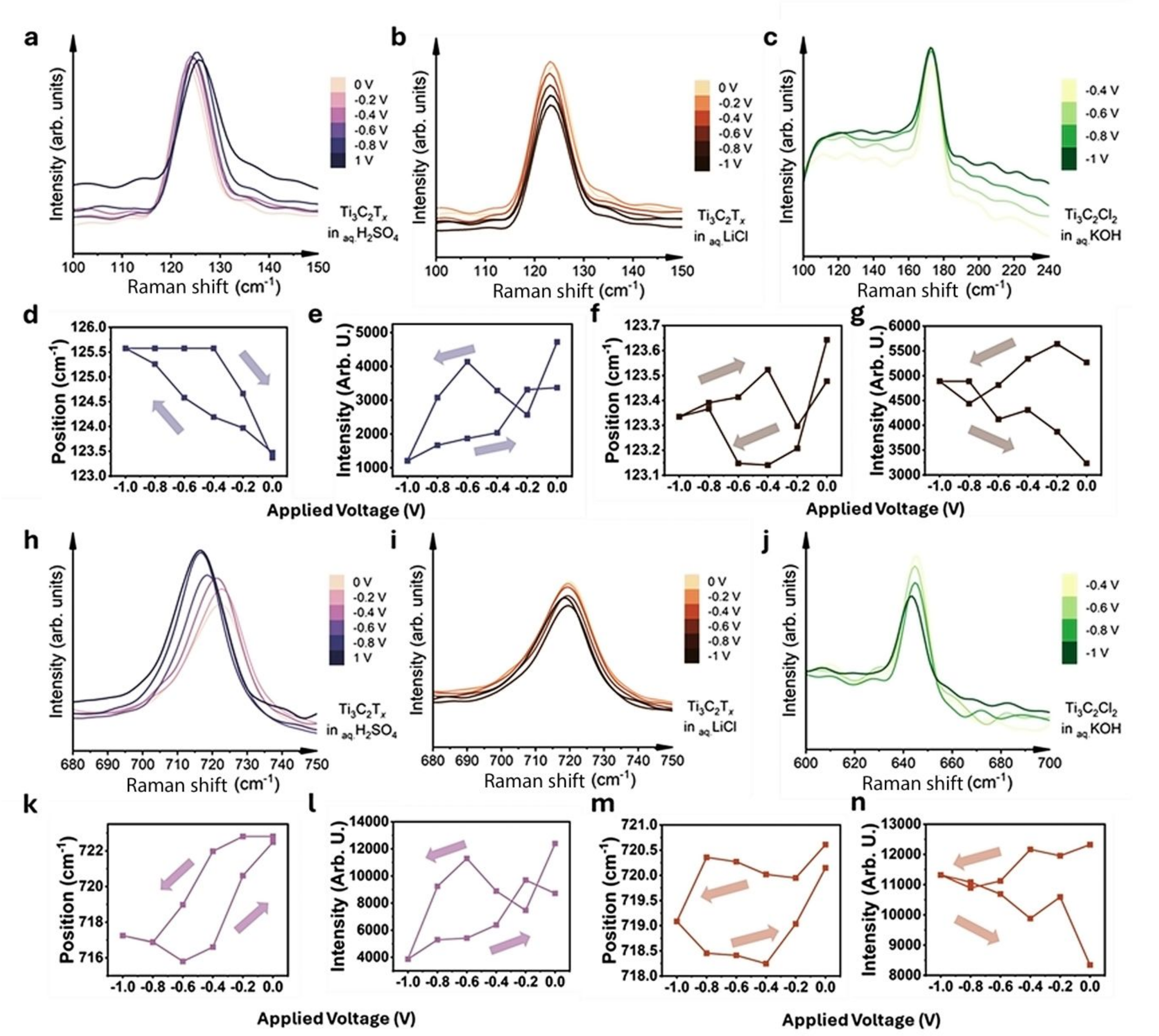


**Figure S6.** Raman spectra peak position and intensity changes. $E_g$ (Ti, C, $T_x$) mode in a) $H_2SO_4$ electrolyte shows the most significant peak position shift, while in b) LiCl electrolyte only minor position shift and intensity variations are observed b). c) $A_{1g}$ (Ti, C, Cl) mode shows no peak position shift in KOH electrolyte. Raman peak position and intensity changes of $E_g$ (Ti, C, $T_x$) mode shown for (d-e) $H_2SO_4$ electrolyte and (f-g) LiCl electrolyte upon cycling from 0 V to -1 V and back. $A_{1g}$ (C) mode shows the largest peak position shift in h) $H_2SO_4$ electrolyte system, and minor peak position shifts in i) LiCl and j) KOH electrolyte. Raman peak position and intensity changes of $A_{1g}$ (C) mode shown for (k-l) $H_2SO_4$ electrolyte and (m-n) LiCl electrolyte upon cycling from 0 V to -1 V and back.

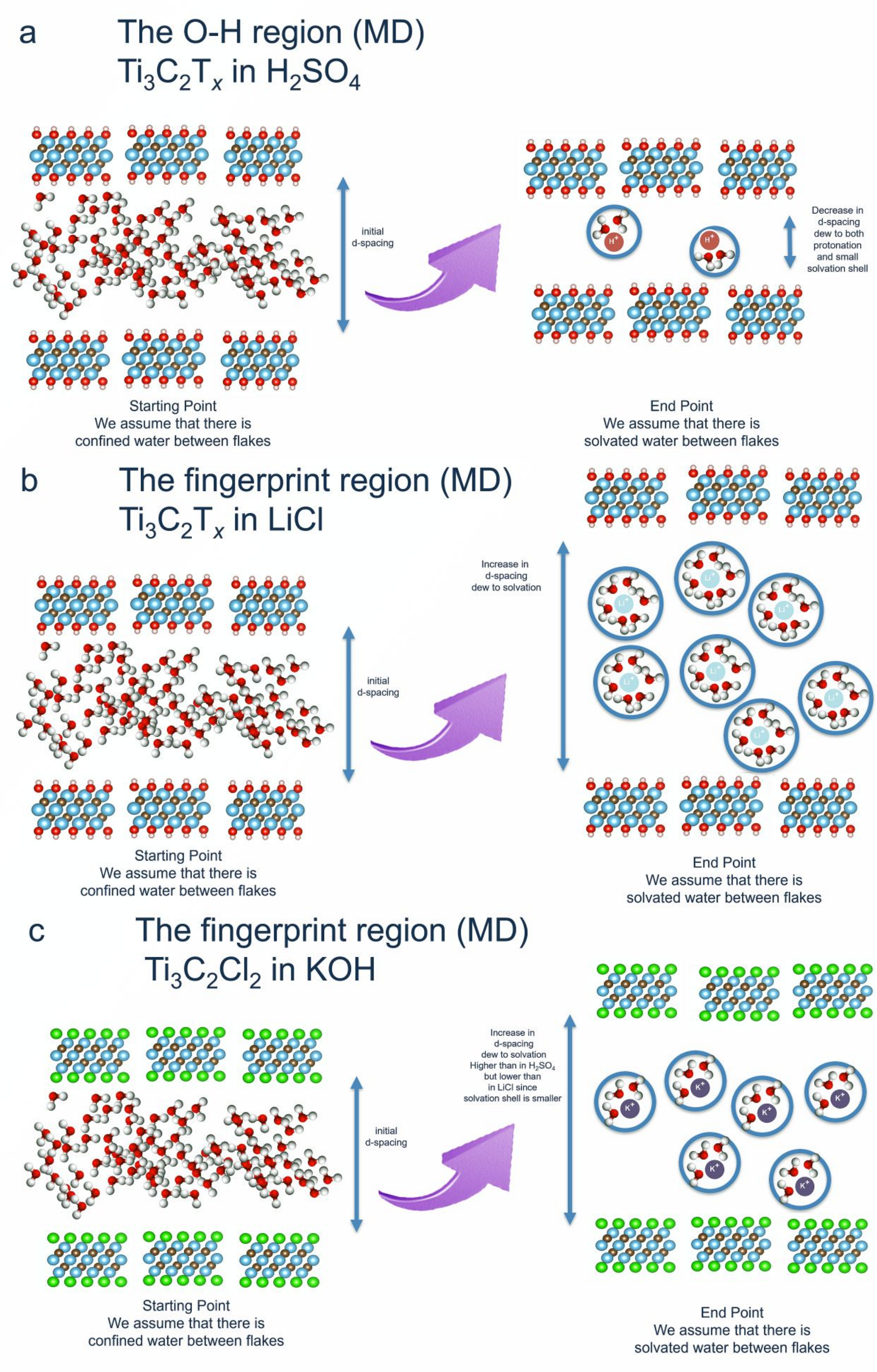


**Figure S7.** Detailed comparison of OH-confined vs. OH-solvated systems for a) $Ti_3C_2T_x$ in $H_2SO_4$, b) $Ti_3C_2T_x$ in LiCl, c) $Ti_3C_2Cl_2$ in 6 M KOH
